# Chemical Reaction Networks’ Programming for Solving Equations

**DOI:** 10.3390/cimb44040119

**Published:** 2022-04-14

**Authors:** Ziwei Shang, Changjun Zhou, Qiang Zhang

**Affiliations:** 1 Key Laboratory of Advanced Design and Intelligent Computing, Ministry of Education, School of Software Engineering, Dalian University, Dalian 116622, China; shangziw@126.com; 2 College of Mathematics and Computer Science, Zhejiang Normal University, Jinhua 321004, China; zhou-chang231@163.com

**Keywords:** molecular programming, chemical reaction networks, chemical dynamics, biological molecular calculations

## Abstract

The computational ability of the chemical reaction networks (CRNs) using DNA as the substrate has been verified previously. To solve more complex computational problems and perform the computational steps as expected, the practical design of the basic modules of calculation and the steps in the reactions have become the basic requirements for biomolecular computing. This paper presents a method for solving nonlinear equations in the CRNs with DNA as the substrate. We used the basic calculation module of the CRNs with a gateless structure to design discrete and analog algorithms and realized the nonlinear equations that could not be solved in the previous work, such as exponential, logarithmic, and simple triangle equations. The solution of the equation uses the transformation method, Taylor expansion, and Newton iteration method, and the simulation verified this through examples. We used and improved the basic calculation module of the CRN++ programming language, optimized the error in the basic module, and analyzed the error’s variation over time.

## 1. Introduction

Since biomolecules can solve complex problems [1], biomolecular computation intends to perform algorithms and calculations through synthetic biochemical systems. More important is how to realize the computational models generated by biochemical processes through programming [2]. The realization of computational models in CRNs that use biomolecules as the substrates is primarily analogous to traditional engineering processes or systems with computational capabilities. The components in all calculation models are plug-and-play chemical reaction network calculation models in a test tube solution [3]. An important issue is abstracting the basic reaction module [4] and controlling the calculation order [5,6,7]. Excellent work has been performed in the realization of computing and programmability [8,9,10,11,12,13]. Based on the characteristics of different molecules, people have developed a system that can realize logic operations [14,15,16,17] and a calculation model with reprogrammable execution algorithms [18]. Among them, DNA molecules can undergo branch migration and strand displacement reactions due to their base complementation [19,20] and can realize storage structures through programming [21,22,23,24] and accomplish signal transmission [25]. The logic gates constructed based on the above characteristics of DNA molecules can realize the construction of large-scale cascade circuits [26] and neural networks [27]. There is also the construction of cascade circuits by designing arithmetic gate modules to realize the network of analog function calculation [28] and the simulation of polynomial functions [29].

Nevertheless, the gate module structure that uses the circuit to realize the calculation limits the processing of the operation steps. For example, the output of each two-input gate module needs to be cascaded with the next gate module. This calculation model’s shortcomings that depend on the circuit characteristics are reflected primarily in the implementation of simulation functions [28,29] and equation solving calculation [30]. For example, Song and Zou needed operation gates to build each item and then cascaded them to realize the simulation when constructing polynomials [29,30]. Even if Salehi adopted Horner’s law to relatively reduce the repetition of calculations [28], it could not reduce the repeated input coefficient parameters required when building each item and the significant truncation error caused by the limitation of the cascade structure. In Zou’s paper [30], due to module limitations, the design of analytical solutions of equations could not be achieved, leading to repeated techniques in solving similar equations. Given the problems in the above calculations, we focused on using a formal, circuit-free corresponding design method to overcome these.

Implementing a circuit-free structure design in the CRNs is based on the chemical reaction networks being an abstract calculation model, a programmable chemical controller that can be realized in the CNRs with mass effect [31]. There has been much excellent work in mapping ordinary differential equations’ (ODEs) system models abstracted from different computing systems to realize the corresponding functions through chemical reaction networks. Buisman synthesized conceptual networks that carried out elementary mathematical operations to realize the calculation of algebraic functions [32]. In Cardelli’s paper, Cardelli discovered the correspondence between the linear circuit function and the chemical reaction network [33]. Ge designed a circuit-less chemical reaction network to realize the logic design of the corresponding Karnaugh map [34]. Vasic first developed the CRN programming language—the CRN plus plus (CRN++) language [35]. The above controllable and programmable chemical reaction networks use DNA molecules as general reaction substrates because DNA molecules can realize arbitrary coupling chemical reaction networks [6,36].

This paper proposes a corresponding design method for solving nonlinear equations in the chemical reaction network. We used a formal, circuit-free corresponding design method to achieve the calculations. We designed discrete and analog algorithms and used these algorithms to fit equations with and without analytic solutions. We designed and solved equations that the previous calculation structure could not achieve, such as exponential equations, logarithmic equations, and the simplest trigonometric equations. Given the limitation of the gate structure of the circuit to realize the calculation, we used a module more in line with the arithmetic logic in the solution process so that we could simulate the algorithm through the combination of basic arithmetic modules in the CRNs. The simulation algorithm in the chemical reaction network realizes the simulation of the polynomial function, which reduces the cost of constructing each term of the gate module, and the simulation of the expansion of the function into a Taylor polynomial also allowed us to solve the equation without an analytical solution by the Newton iteration method.

Furthermore, for previous methods of repeatedly constructing equations rather than directly constructing analytical solutions, we simulated analytical solutions through discrete algorithms so that the same type of equation solving reduced the repetitive design. When solving the same type of equation, since the analytical solution is the same, it is only necessary to change the concentration according to the coefficient without rebuilding it. The advantage of the discrete algorithm is that it can connect multiple chemical reaction network function modules, making it possible to effectively connect them according to the order of their calculation when simulating the analytical solution. This article used CRN++ to program to achieve the solution of all equations. At the same time, because reducing errors is also the focus of CRNs’ design, we also optimized the basic calculation modules in CRN++ in the solution design. We combined and designed more accurate calculation models by improving the basic modules.

The rest of this article is organized as follows. Section 2 gives a brief description of the calculation principle of the equation solving and mapping to the chemical reaction network and the reaction substrate, and we improved the algorithm of the division module in CRN++. Section 3 presents the process of solving the three types of equations corresponding to the designed discrete algorithm and simulation algorithm and the results of the simulation realization. Moreover, we compared the error of the improved algorithm and analyzed the simulation algorithm’s error change when simulating the function. Section 5 summarizes the full text.

## 2. Materials and Methods

**Figure 1:**
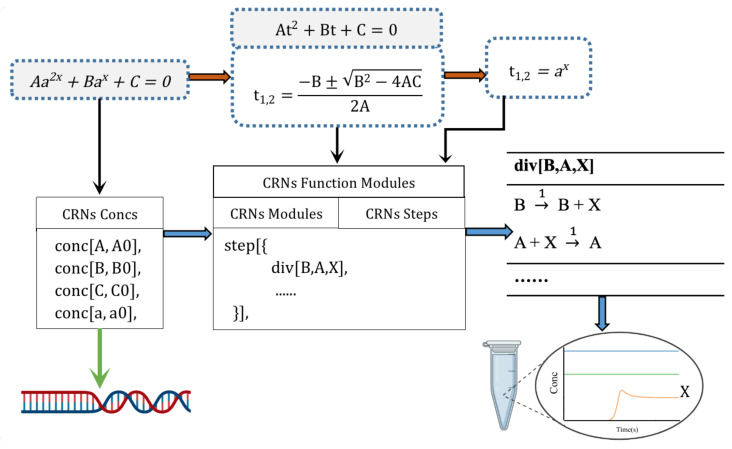
The basic design framework principle. The solid red line represents the mathematical steps of solving the equation, and the solid black line represents the mapping in the design of the CRNs. The solid blue line represents the process of the CRNs compiled, and the solid green line represents the corresponding molecular substrate in the CRNs. The gray background does not require construction steps, so only the parameters are extracted.

We introduce the computational methods and principles of solving nonlinear equations in CRNs, as well as the substrates in the reactions. First, we briefly describe the process of solving calculations in the CRNs, the basic calculation modules used in the computing, and the composability of the time phase between the modules. Figure 1 show the corresponding solution process of the equation with the analytical solution. For the equation to be solved, its coefficients need to be mapped to the concentration of reactive species.

We designed and solved the solution scheme to realize the substitution function and map the solution steps to different functional modules. Then, we analyzed the basic calculation modules that need to be used in each function module, such as addition, subtraction, multiplication, division, and the division of comparison and judgment. Finally, we considered that the calculation module needs to be divided into several sequential steps.

All the chemical reaction equations in the function module corresponding to the solving steps can generate CRNs that realize the solution, and the concentration of the product expresses the root of the equations. This network uses DNA molecules as the substrates because DNA molecules can be coupled to any chemical reaction, so we did not consider the design of DNA molecules here. In the simulation calculation, we set all chemical reaction rates as *k* = 1 and omitted them in the following reaction equations.

### 2.1. Basic Modules

The calculation model for solving the equation uses basic operation modules, such as div[B,A,X] representing division, and other operations for comparison and judgment [32]. Each operation module contains several chemical reaction equations, and the calculation order ensures mutual isolation through clock species regulation. Each function module includes the integration of these basic operation modules [35]. The basic principle is explained by division. The CRNs transformed from the algebraic function operation of division are expressed as Equations (1) and (2):

(1) B→B+X

(2) A+X→A

**Figure 2:**
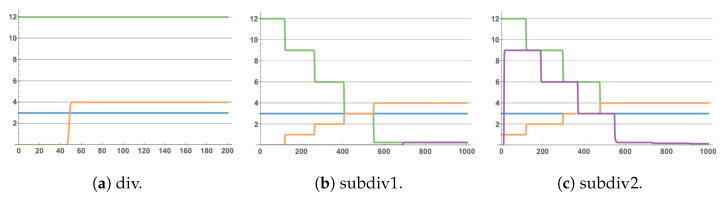
Dynamic simulation of the solution results of solving AX=B of the three methods, (**a**) where A(0)=3 (blue), B(0)=12 (green), solution result of X=4 (orange); (**b**) the remainder is r=0.259458 (purple); (**c**) the remainder is r=0.1472 (purple). The abscissa represents the time unit is seconds.

The reaction environment was carried out in a fully mixed molecular solution that is continuous in time and constant in the state space. In the CRNs, the change of each species can be expressed by a system of ordinary differential equations [35]:

(3) $$\dot{S}=\sum_{\forall rxn\in CRNs}k\cdot \mathrm{change}\left(s\right)\cdot \prod_{\forall R\in \mathrm{reactions}\;(rxn)}{\left[R\right]}^{m}\left(t\right)$$

where $\;\dot{S}$ is the differential equation of the concentration of the product over time, *k* is the rate of the chemical reaction equations, change(s) is the net change of the reactant, *R* is the reactant, and *m* is the number of repetitions of the reactant. In summary, the equations in the chemical reaction network of the division can be transformed into ODEs: $\;\dot{A}=0$,$\;\dot{B}=0$, $\;\dot{X}=\left[B\right]\left(t\right)-\left[A\right]\left(t\right)\cdot \left[X\right]\left(t\right)$. The expression of ODEs also depends on the reaction finally reaching a unique steady-state. When the steady-state is reached, $\;\dot{X}=0$, such that a basic module can complete the solution of the one-dimensional linear equation because the A and B species are used as coefficients to react. [A](t)[X](t)=[B](t), to more concisely show the relationship between species changes, in the rest of this article; we omitted the explicit dependence on time, writing the equation AX=B instead of [A](t)[X](t)=[B](t). There is no change before and after, so the assignment of the coefficient is [*A*](0), [*B*](0), which is the initial concentration of the species. The input data selected in the simulation were *A* = 3, and *B* = 12, and the calculation result is shown in Figure 2a. This operation of preserving input values makes some input species equivalent to catalysts. We can also see that each changed species in the figure occurs from one step to the next. During the simulation, we only show the final steady-state of the species after each step. As can be seen from the results in Figure 2, in simulating the change of species concentration with time, we did not consider the change of species concentration in each step, but only show the concentration evolution at the end of each step. Each step refers to a calculation step that requires at least three clock species, while only one clock species participates in the calculation. The three clock species form a chemical oscillator to isolate this calculation from the following calculation. All reactions in the chemical reaction network have a clock species as reactants in this calculation step. The result chart shows only a straight line in a calculation step, that is it only shows the time required for this calculation step, and the change of concentration is displayed after the settlement of one step, that is it needs to reach the steady-state. Therefore, the simulated concentration will change vertically after each calculation step.

The ODEs composed of all reactants and products are the critical bridge to transforming algebraic functions into CRNs. The prerequisite for realizing the corresponding relationship is whether all the reactions in the chemical reaction network can reach a unique stable state. The calculation model requires that only when the chemical reaction equilibrium is reached, the calculation module can correspond to the corresponding ODEs. Note that the chemical reaction equation in the reaction module, Equation (1), does not obey the law of conservation of mass. It is an abstract chemical reaction equation to delete irrelevant non-catalytic reactants to design the algebraic function module better [6].

The division module in CRN++ can use subtraction replacement to filter to accumulate part of the quotient error in the operation, obtaining the quotient and remainder through subtraction. The advantage of this method is reflected in the solution of the quadratic equation of one unknown below. This article improved the implementation of division in the basic module used. In the CRN++ module introduction, examples of using division and converting to subtraction operations [35] are already given, as shown in Figure 2b. Algorithm 1 is our improved calculation step, and the simulation results are show in Figure 2c. Shown in Figure 2 is the simulation results of the three ways to achieve division. The development of the error analysis is in Section 4.
**Algorithm 1:** subdiv2 Reaction.   **Data:** Concentration of species $\;\left[S_{i}\right]\left(0\right)\in R,S_{i}=\left[A\right]\left(0\right),\left[B\right]\left(0\right)\ldots$   **Result:** [Quot](t), [Rema](t). 1 **while**
*not at end of the simulation time*
**do** 2  Rema←B−A 3   **if**
Rema≥A
**then** 4     QuotNext←Quot+1 5     B←Rema 6  Quot←QuotNext 7  **end**

### 2.2. Composable Basic Modules

The transfer of parameters between different functional modules in CRNs is based on the principle of DNA strand replacement. Each step in the same function module, the isolation, and the execution of the basic arithmetic modules is controlled by the chemical reaction oscillator generated by the clock species. All reactions reach a state of equilibrium and exponentially converge to ensure that the product’s output can be used as the input of the next module.

The harmonious combination of various modules and the independence of each are key to ensuring that the CRNs realize calculations. For example, the function of division can be expressed as step[div[B,A,X]], step[ld[B,X]]; these two calculation modules need to be isolated in two steps to complete the calculation. In the combination, we used the method of retaining the reactants. Suppose the design directly performs the chemical reaction of the input species since the reactions in the CRNs are carried out in parallel. In that case, the input of the products from the previous module to the input of the next module will affect the module’s performance. Equalization will cause the calculation modules to be uncombinable [35].

**Figure 3:**
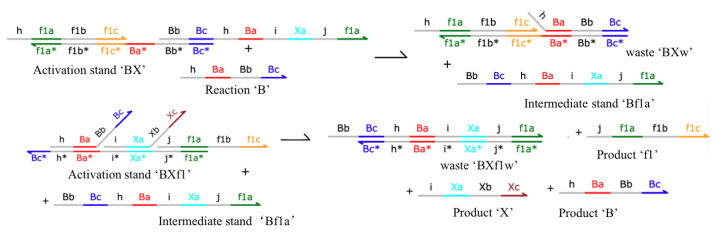
A list of the DNA reactions in B→B+X. The DNA domain of the color part is assumed to represent a unique DNA sequence. It is assumed that a domain can be bound to its complement, but cannot interact with any other domain in the system. It is also a part that can branch and migrate.

DNA strand displacement technology plays a role in controlling signal transmission. Figure 3 shows a series of reactions in which DNA molecules fit the chemical reaction Formula (1). We enumerate how the first formula in Equation (1) can be expressed as Equations (4) and (5):

(4) BX+B→BXw+Bf1a

(5) BXf1+Bf1a→BXf1w+f1+X+B

To simplify the model, we assumed that the binding reaction is irreversible in the simulation. We call the Ba* domain in *BX* a toehold. In Equation (4), the Ba domain in *B* hybridizes with the Ba* domain in *BX*, where the asterisk indicates the complex domain. First, the toehold will automatically match according to the principle of base complementation, where the Bb and Bc domains in *B* will compete with the Bb and Bc domains in *Bf1a*. In the end, strand *B* will replace strand *Bf1a* and complete the strand displacement reaction. By designing the sequence of different base sequences in the toehold, reactions can generate different strand replacement reactions. The replaced single strand can be used as the input of other modules to distinguish between different modules and transmit different signals. Strand *B* is both a reactant and a product, which is regarded as a catalytic reaction among all interacting species. In the CRN simulator, only the steady-state after each step is described, so the concentration of some input species remains macroscopically unchanged.

## 3. Results

This part mainly describes three kinds of equations: logarithmic equations, exponential equations, and the simplest trigonometric equations to solve the process and the results. Among them, the solution of the two exponential equations corresponds to two methods, respectively. One is to use the analytic method to obtain the roots of the equation through the analytical solution. At the same time, the quadratic equation in one unknown uses the method of substitution to find its solution first and then solve the exponential equation. This design shows that using discrete algorithms can solve complex equations that could not be designed and expressed before. The other is to use Newton’s method to calculate the result after each iteration by developing the corresponding Newton iteration formula in the chemical reaction network. We uses real-valued (analog) algorithms to solve the other two types of equations.

The solution of an equation may require multiple function modules. If a function module has been active until the end of the simulation time, we need to divide it into different modules. The state of such activation is in two situations. One is the existence of the CMP module [35], which generates branch control by comparing different concentration values, and the other is a loop assignment statement that the loop will cause. The above implementation principles are because the chemical oscillator controlling the steps continues to oscillate under certain conditions. This will cause errors in the subsequent addition of calculation steps in the same function module. To ensure the smooth progress of the calculation, we must ensure that a function module has only one CMP module. There is no step relationship between different function modules. In other words, we isolated different function modules by adding clock species and used a group of clock species in the same function module. The clock species are regulated by the step. The DNA strand replacement technology ensures that the specific species are the output of the upstream function module and the input of the downstream function module. The correlation between the upstream function module and the downstream function module is that the concentration of the species output by the upstream function module is the initial concentration of the input species of the downstream function module, so this requires that the downstream function module calculation input cannot be negative.

Algorithm 2 represents the processing of the negative input after one step in the CRNs. Such a pseudo-concentration can be output after the calculation reaction of changing the negative value through subtraction, addition, squaring, etc., in the calculation.
**Algorithm 2:** Negative numbers in steps.    **Data:** Concentration of species $\;\left[S_{i}\right]\left(0\right)\in R,S_{i}=\left[A\right]\left(0\right),\left[B\right]\left(0\right)\ldots$    **Result:** H(t) 1  The value H from the end of one step to the next step; 2  **if** H>0
**then** 3    Continue to the next step of calculation. 4  **else** 5    H=0 6  **end**

### 3.1. Solving the Exponential Equations

We solved two different types of exponential equations. The first was an equation with an analytical solution, $\;Aa^{2x}+Ba^{x}+C=0$. The second was a nonlinear equation without an analytical solution, $\;x^{3}e^{x}-2=0$.

For the equation with the analytical solution, $\;Aa^{2x}+Ba^{x}+C=0$, we constructed its execution order in the CRNs by the discrete algorithm. The concentration of species corresponding to the root value of the univariate quadratic equation is transferred as an input parameter to the function module for calculating the exponential equation. This design completes the corresponding element transformation method. We used Newton’s method to obtain the solution through iterative calculations for the second nonlinear equation $\;x^{3}e^{x}-2=0$ with no analytical solution. The chemical oscillator can ensure that these reactions can proceed smoothly.

**Figure 4:**
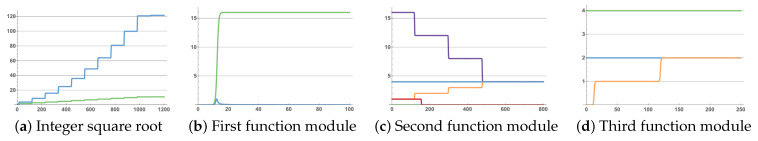
Dynamic simulation of the results of solving $\;2a^{2x}-5a^{x}-12=0,a=2$: (**a**) solving the square result of the integer, corresponding to the solution of $\;\sqrt{\Delta }$,$\;\sqrt{\Delta }=11.0318$ (green); (**b**) solution result of two values of $\;H_{1,2}$, $\;H_{1}=16.0318$ (green), $\;H_{2}=0.00103034$ (blue); (**c**) solution result of $\;t_{1}=4$ (orange), $\;t_{2}$ (red); (**d**) solving the exponential equation to obtain x=2.00001 (orange). The abscissa represents the time unit is seconds.

**Figure 5:**
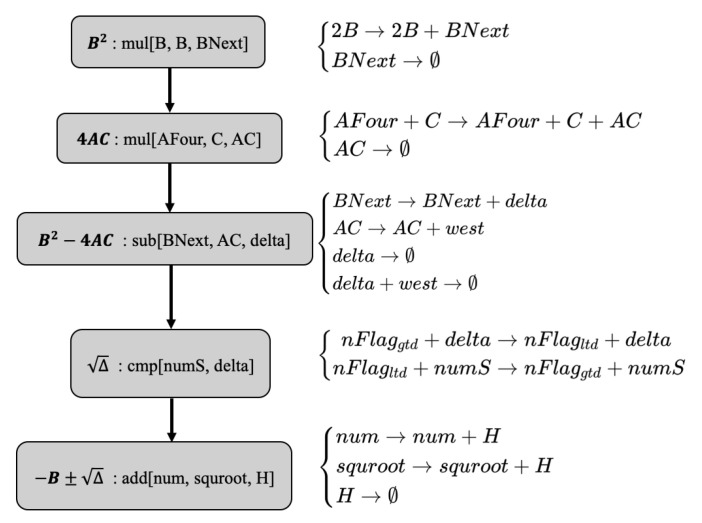
CRNs’ calculation module corresponds to the main calculation steps in the first function module.

To solve the equation $\;Aa^{2x}+Ba^{x}+C=0$, we set $\;t=a^{x}$(t>0) and obtained $\;At^{2}+Bt+C=0$, which contains the solution of two types of equations: The first is the solution of the quadratic equation of one variable corresponding to two function modules in the CRNs. The resolution of the exponential equation corresponds to one function module in the CRNs. A total of three function modules are required to achieve the solution. Figure 4 shows the phased results obtained by the three function modules under specific values, and we solved the open square root using the CMP module. It is precisely because of the existence of this CMP module that our solution before substitution needs to be divided into two modules. First, we designed the calculation module corresponding to the analytical formula. The first function module corresponds to the calculation of $\;H_{1,2}=\left(-B\pm \sqrt{\Delta }\right)$. Figure 5 shows the step design of obtaining the chemical reaction equation of H. Because the square root calculation in this function module uses the CMP module, it is used as a separate function module to ensure the calculation sequence. $\;t_{1,2}=\frac{H_{1,2}}{2A}$ corresponds to the second function module. According to Algorithm 2, we dealt with the case of negative numbers in the calculation. We used Algorithm 2 in the division calculation, and the purpose was to filter to calculate the accumulated error.

**Figure 6:**
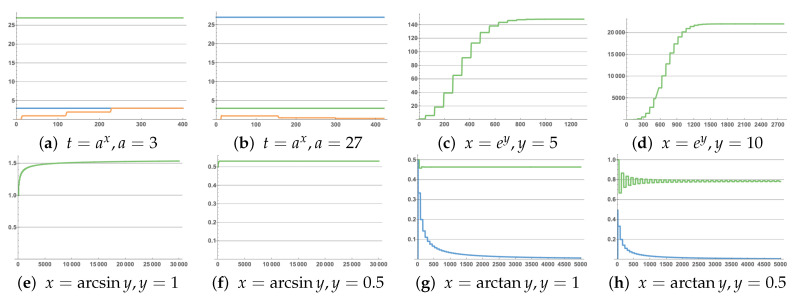
Solution result graph: (**a**) where *a* = 3 (blue), *t* = 27 (green), *x* = 3.00038 (orange); (**b**) where *a* = 27 (blue), *t* = 3 (green), *x* = 0.333333 (orange); (**c**) where *x* = 148.413 (green); (**d**) where *x* = 22,026.5 (green); (**e**) where *x* = 1.54313 (green); (**f**) where *x* = 0.531584 (green); (**g**) where *x* = 0.781827 (green) and absolute value of coefficient (blue); (**h**) where *x* = 0.463648 (green) and absolute value of coefficient (blue). The abscissa represents the time unit is seconds.

The third function module is to solve $\;t=a^{x}$. We used a discrete algorithm (Algorithm 3) to solve the problem and compared the output value t of the second function module with the value of the product of a. We limited the x that we sought to only be a positive integer or the reciprocal of a positive integer, and it can only solve the case where a>1. These two situations occur respectively in the case of t>a and t<a. Corresponding to the simulated values of these two cases, *a* = 3, *t* = 27, *x* = 3.00038; *a* = 27, *t* = 3, *x* = 0.333333, Figure 6a,b shows the results.

The solution of the equation needs to go through the above three function modules. The input data we chose to use in the simulation were *A* = 2, *B* = −5, *C* = −12, and *a* = 2, and the results are shown in Figure 4. We obtained $\;H_{1}$ = 16.0318. Using the div module, the result was $\;t_{1}$ = 4.00795, and using the subdiv2 module, the result was $\;t_{1}$ = 4.

For the equation $\;x^{3}e^{x}-2=0$, we used Newton’s iteration method to approximate the linearization of the nonlinear equation. Then, we set $\;f\left(x\right)=x^{3}e^{x}-2$, and then, $\;f^{\prime }\left(x\right)=\left(3x^{2}+x^{3}\right)e^{x}$. According to the iterative formula of Newton’s method:

(6) $$x_{n+1}=x_{n}-\frac{f\left(x_{n}\right)}{f^{\prime }\left(x_{n}\right)}$$

and then, sorted out by Newton’s iterative formula:

(7) $$x_{n+1}=x_{n}-\frac{x_{n}^{3}e^{x_{n}}-2}{\left(3x_{n}^{2}+x_{n}^{3}\right)e^{x_{n}}}\;n=0,1,2,\cdots$$

The solution to $\;e^{x_{n}}$ here uses the simulation Algorithm 3 in the next section, taking the initial value $\;x_{0}=1$. Table 1 lists the iteration results.

**Table 1:** Comparison of the calculated value in CRNs and the exact value.

*n*	CRNs Computed	Exact
0	1	1
1	0.933940	0.933940
2	0.925244	0.925600
3	0.921332	0.925479
4	0.921607	0.925479

In Table 1, *n* represents the number of iterations. The first iteration in the chemical reaction network can be accurate to six digits after the decimal point. After four iterations, it can be accurate to four digits after the decimal point. The convergence speed in the chemical reaction network was also faster. However, the accuracy decreased as the number of iterations increased.

### 3.2. Solving the Logarithmic Equations

We solved the logarithmic equation as: $\;\log_{n}x=b$; the most direct way is to convert it into an exponential function: $\;x=n^{b}$; its solution in the integer range was achieved by Algorithm 3. However, when solving lnx=b, we used real-valued (analog) algorithm calculations. In the CRNs, we approximated infinite computations through assignment loops. As long as the simulation time does not stop the reaction, it can continue forever, avoiding truncating the McLaughlin series. Here, we calculated $\;x=e^{b}$, which is equivalent to a simulation of the function $\;f\left(y\right)=e^{y}$. We needed to expand f(y), using Taylor’s formula:

(8) $$f\left(y\right)=\sum_{n=0}^{\infty }\frac{f^{\left(n\right)}\left(y_{0}\right)}{n!}{\left(y-y_{0}\right)}^{n}$$

When $\;y_{0}=0$, the Maclaurin expansion of $\;e^{y}$ can be obtained:

(9) $$e^{y}=\sum_{n=0}^{\infty }\frac{y^{n}}{n!}=1+y+\frac{y^{2}}{2!}+\frac{y^{3}}{3!}\cdots$$

Algorithm 3 can simulate its value through the input and repeated use of the basic calculation module. The calculation of the simulation function obtained high accuracy results in CRN++. The input data selected in the simulation were: *b* = 5; the calculation result was *x* = 148.413, which can be accurate to the last three decimal places; when *b* = 10, the calculation result was *x* = 22,026.5; the result is shown in Figure 6c,d.
**Algorithm 3:** Exponential Reaction.   **Data:** Concentration of species $\;\left[S_{i}\right]\left(0\right)\in R^{+},S_{i}=\left[a\right]\left(0\right),\left[t\right]\left(0\right)\ldots$ [aEqu](0) =     [a](0).   **Result:** [x](t). 1 **while**
*not at end of the simulation time*
**do** 2   aNext←a×aEqu 3   xNext←x+1 4    **if**
a<t
**then** 5      a←aNext 6      x←xNext 7 **end**

### 3.3. Solving the Simplest Trigonometric Equations

We used simulation algorithms in the CNRs to solve the two simplest types of trigonometric equations, sinx=b, tanx=c. We did not consider the solution set, found the corresponding inverse function by limiting the numerical range of the unknown number, and found the solution by simulating the approximate polynomial of the inverse function through the simulation algorithm. The chemical oscillator is a series of reactions that the clock species can continuously convert. The use of the chemical oscillator allows the simulation algorithm to be repeated in theory until the simulation time stops. This avoids that the gate design alone cannot be physically infinite and reduces the repetitiveness of the construction. The simulation algorithm shows its calculation ability in the solution of lnx=b.

The realization of the solution method we proposed must be within the given numerical range, sinx=b, where $\;x\in [0,\frac{\pi }{2}]$, within this value range. We can convert it into the inverse function on the corresponding interval x=arcsinb, and we set f(y)=arcsiny, y∈[0,1] and expanded f(y) according to Taylor’s formula Equation (8); when $\;y_{0}=0$, we can obtain the Maclaurin expansion of Equation (10):

(10) $$\begin{array}{cc} \arcsin y= & \sum_{n=0}^{\infty }\frac{(2n-1)!!}{(2n)!!}\cdot \frac{y^{2n+1}}{2n+1}=y+\frac{1}{2}\cdot \frac{y^{3}}{3}+\\ & \frac{1\cdot 3}{2\cdot 4}\cdot \frac{y^{5}}{5}+\frac{1\cdot 3\cdot 5}{2\cdot 4\cdot 6}\cdot \frac{y^{7}}{7}+\ldots \end{array}$$

The input data selected in the simulation were: *y* = 1; the calculation result was *x* = 1.54313, which can be accurate to the last three decimal places; when *y* = 0.5, the calculation result was *x* = 0.531584; the result is shown in Figure 6e,f.

The realization of the solution method we proposed must be within the given numerical range, tanx=c, where $\;x\in [0,\frac{\pi }{2})$, within this value range. We can convert it to the inverse function on the corresponding interval x=arctanc, and we set f(y)=arctany, y∈[0,+∞), expanded f(y) according to Taylor’s formula Equation (8); when $\;y_{0}=0$, we can obtain the Maclaurin expansion of Equation (11):

(11) $$\begin{array}{cc} \arctan y & =\sum_{n=0}^{\infty }\frac{{\left(-1\right)}^{n}}{2n+1}y^{2n+1}\\ & =y-\frac{1}{3}y^{3}+\frac{1}{5}y^{5}-\frac{1}{7}y^{7}+\cdots \end{array}$$

The implementation steps of Equation (11) in the CRNs are as follows Algorithm 4.
**Algorithm 4:** Arctan Reaction.    **Data:** Concentration of species $\;\left[S_{i}\right]\left(0\right)\in R,S_{i}=\left[y\right]\left(0\right),\left[B\right]\left(0\right)\ldots$ [deno](0) = 1,      [oneNegative](0) = −[y](0), [yEqu](0) = [y](0), [arcTan](0) = [y](0)    **Result:** [arcTan](t), [coef](t).  1 **repeat**  2   step : denoNext←deno+2  3    coef←1/denoNext  4    ySqu←y×yEqu  5    yNext←ySqu×yNegative  6    term←coef×yNext  7    arcTanNext←arcTan+term  8   step :y←yNext  9    arcTan←arcTanNext 10    deno←denoNext 11 **until**
*end of the simulation time*;

We need to pay attention to the execution order of the critical steps. For example, the assignment statement must be after the calculation statement. It must be in various steps to ensure that the calculation response is separated from the assignment response in time to confirm the order of the steps.

The input data selected in the simulation were: *y* = 1; the calculation result was *x* = 0.781827, which can be accurate to the last three decimal places; when *y* = 0.5, the calculation result was *x* = 0.463648; the result is shown in Figure 6g,h.

### 3.4. Error Evaluation

There are many reasons for errors in the calculation model designed in the CRNs, including the accumulation of errors caused by the collection of the calculation steps, the inaccuracy of the product concentration fitting to the final value, and the calculation of the conversion of the CRNs into ODEs in the basic principle. The reaction needs to reach a stable state in an infinite time, but this is impossible to achieve, so that it will cause inevitable errors during the simulation. For the generation of the first error, the most direct way to reduce the error when designing the calculation model is to minimize the error of the basic calculation module. The implementation method was to reduce the error by replacing different modules and adjusting the reaction steps.

In Table 2, we compare the quotient errors in the three modules in Figure 3 that implement the division function in the CRNs. We can see that the total error of the single-step division module div was larger than the accumulated error of the multi-step subtraction, which is one of the reasons why we chose to use removal instead. However, the main reason is reflected in solving the quadratic equation of one variable. After a series of calculation steps, the subtraction will filter out a part of the error generated by the dividend and filter it into the remainder.

In Table 3, we compare subdiv1 and subdiv2, comparing the error generated on the remainder and the dividend, where subdiv2 is our improved module. In Table 2, we can see that subdiv1 and subdiv2 had almost no noticeable difference in the calculated error of the quotient. Still, there was a pronounced difference in the error of other values, such as the remainder and the dividend. The above shows that in addition to the inevitable errors, the errors generated by the calculation model of the design CRNs can be minimized by combining different timing steps of different design algorithms.

**Figure 7:**
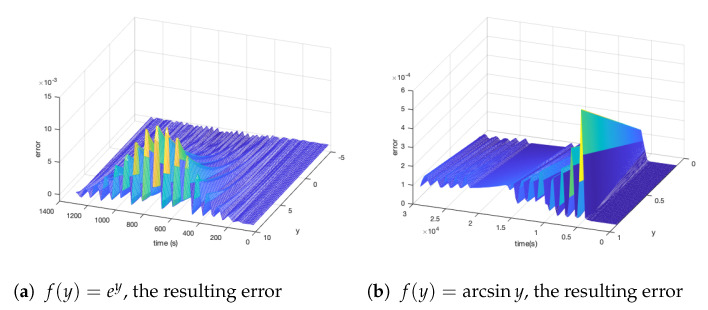
Errors (**a**) and (**b**) generated by simulation algorithms over time and value growth. The *y*-axis represents the value of *y*, the *x*-axis represents the simulation time length, and the *z*-axis represents the error between the *x* calculated in the CRNs and the actual solution.

We analyzed the error of $\;f\left(y\right)=e^{y}$ and f(y)=arcsiny simulated numerical calculations (Figure 7). According to the simulation time and the increase of the y value, we show the error change. The error value is Statistics at the end of each step. We can see that the error of the simulation values of the two simulation functions gradually increased with the increase of the y value. $\;x=e^{y}$. Almost all response errors were concentrated in 400–1000 s. Through Figure 6c,d, we can see that this period was a stage where the simulation value rose rapidly. After 1000 seconds, the change of the numerical simulation tended to be stable. For f(y)=arctany, the calculation had a significant error at the beginning. Almost all the response errors concentrated on the first 15,000 seconds, that is a greater error was generated when the value involved in the response changed significantly. In the error of the simulation, we can also see that the error will not always change with the increase of time. The reason is that the numerical change calculated with the rise of the simulation time will be more minor and the result will be more stable.

Table 4 shows the species used in the chemical reaction network to solve the equation and the reaction scale.

**Table 4:** Size of CRNs.

Program	All Species	Clock Species	Reactions
Exponential	35	12	41
Logarithmic	15	6	22
Arcsin	37	9	39
Arctan	27	6	26

## 4. Discussion

In the section on error analysis, we analyzed the error generated by the simulation. In the actual calculation, when the CRNs are realized through DNA coupling, there may be significant errors, especially when the number of molecules is relatively small; the small number of molecules will lead to non-negligible fluctuations and the discrete nature of the molecular concentration, which may change the frequency of the reaction events of each chemical substance. Molecular fluctuations may dominate the dynamics, and these molecular fluctuations seem to have many significant biological consequences. Therefore, the deterministic model used in our simulation does not always accurately describe the chemical dynamics of such systems because the statistical average cannot explain the molecular fluctuations. In order to simulate the molecular fluctuations, a probabilistic model of stochastic chemical reaction networks is usually needed [37]. To avoid the above problems, when calculating the problem of a small molecular concentration, we will enlarge the number of molecules participating in the reaction in equal proportion to ensure the accuracy of the deterministic model.

## 5. Conclusions

This paper simulated and solved the nonlinear equations that could not be solved before and put forth the algorithm design for solving three kinds of equations in CRNs. All frameworks were circuit free, which overcame the limitations of the modular structure used in the previous work and made it possible to realize the algorithm more in line with mathematical logic rather than circuit logic. The solution of these three kinds of equations can be attributed to the design of the algorithms in the CRNs, which can be achieved only when the previous circuit design logic needs to construct very complex modules. In the construction of polynomials, the previous design can undoubtedly approach infinity like the iterative algorithm, which shows the advantage of no circuit structure in the chemical reaction network in solving computational problems.

The discrete algorithm we designed (including subdiv2, solving one-dimensional quadratic equation, exponential function, Newton iteration method), such as when solving the one-dimensional quadratic equation, overcame the previous problem of solving equations and designing different modules for different module coefficients repeatability [30], such as the difference between positive and negative coefficients, requires creating other calculation modules. When solving two different exponential equations, we also realized the conversion calculation in the chemical reaction network for exponential equations with analytical solutions. We realized the corresponding design of Newton’s iterative method through the discrete algorithm for exponential equations without analytical solutions. More importantly, we used analog algorithms to simulate functions to achieve approximate infinite calculations (including $\;e^{y}$, arcsiny,arctany), which overcame the complex problems of cascading and cascading the circuit of a single gate structure cascade when simulating the Taylor expansion. The complex issues of repetition and cascading of the structure of each item were constructed separately. The problem of significant truncation errors in the previous work was avoided [28,29].

Our work expands the ability of chemical reaction networks to solve equations. The results showed that the use of circuit-free structures is more in line with the logic of algebraic calculations in solving polynomial simulations; secondly, the CRNs achieved a certain degree of programmable and computable work. With the development of more and more molecular programming languages, more and more molecular programming languages will appear and continue to improve. Using some of the CRN simulators also made our calculations more reasonable and allowed more types of analyses. Finally, it proved that the algorithm we designed can simulate the solution of the equation more accurately.

The reduction of error was one of the goals of the CRN calculation model. We used modules with more minor errors in solving numerical modules to replace modules with more significant errors. For example, the most typical one is to use subtraction instead of division. This design part of the error generated in the quotient was filtered out. This showed that even in addition to the inevitable errors, the steps of the control algorithm can still minimize the calculated error.

This paper used the CRN simulator to write the algorithm in the Mathematica software (http://users.ece.utexas.edu/~soloveichik/crnsimulator.html, accessed on 20 December 2021) through the CRN++ language to obtain the simulation results and export the generated data to MATLAB to draw a visual error analysis diagram. VisualDSD was used to draw the DNA graphics in the article.

## Figures and Tables

**Table 2:** Error comparison of quotient.

Program	Average Error	Total Error
div	$\;7.1442\times 10^{-7}$	$\;1.9999\times 10^{-5}$
subdiv1	$\;3.9435\times 10^{-8}$	$\;1.1042\times 10^{-6}$
subdiv2	$\;2.9968\times 10^{-8}$	$\;8.3909\times 10^{-7}$

**Table 3:** Total error of dividend and remainder.

Program	Dividend	Remainder
subdiv1	0.1205	0.2594
subdiv2	$\;1.9535\times 10^{-7}$	0.1472

## Data Availability

The data supporting the results reported here are available from the corresponding author upon request.
